# Relations of hippocampal subfields atrophy patterns with memory and biochemical changes in end stage renal disease

**DOI:** 10.1038/s41598-023-29083-0

**Published:** 2023-02-20

**Authors:** Yuhan Jiang, Bingbing Gao, Mingshuai Li, Yangyingqiu Liu, Yuan Li, Na Liu, Yukun Zhang, Qingwei Song, Xinyu Wen, Jian Jiang, Weiwei Wang, Nan Wang, Yanwei Miao

**Affiliations:** 1 Department of Radiology, The First Affiliated Hospital of Dalian Medical University, No. 222 Zhongshan Road, Xigang District, Dalian, 116011 Liaoning China; 2 Department of Nephrology, The First Affiliated Hospital of Dalian Medical University, Dalian, China

**Keywords:** Neuroscience, Cognitive neuroscience

## Abstract

End-stage renal disease (ESRD) results in hippocampal volume reduction, but the hippocampal subfields atrophy patterns cannot be identified. We explored the volumes and asymmetry of the hippocampal subfields and their relationships with memory function and biochemical changes. Hippocampal global and subfields volumes were derived from 33 ESRD patients and 46 healthy controls (HCs) from structural MRI. We compared the volume and asymmetric index of each subfield, with receiver operating characteristic curve analysis to evaluate the differentiation between ESRD and HCs. The relations of hippocampal subfield volumes with memory performance and biochemical data were investigated in ESRD group. ESRD patients had smaller hippocampal subfield volumes, mainly in the left CA1 body, left fimbria, right molecular layer head, right molecular layer body and right HATA. The right molecular layer body exhibited the highest accuracy for differentiating ESRD from HCs, with a sensitivity of 80.43% and specificity of 72.73%. Worse learning process (*r* = 0.414, *p* = 0.032), immediate recall (*r* = 0.396, *p* = 0.041) and delayed recall (*r* = 0.482, *p* = 0.011) was associated with left fimbria atrophy. The left fimbria volume was positively correlated with Hb (*r* = 0.388, *p* = 0.05); the left CA1 body volume was negatively correlated with Urea (*r* = − 0.469, *p* = 0.016). ESRD patients showed global and hippocampal subfields atrophy. Left fimbria atrophy was related to memory function. Anemia and Urea level may be associated with the atrophy of left fimbria and CA1 body, respectively.

## Introduction

Chronic kidney disease (CKD), characterized by the progressive loss of kidney function, has become a major worldwide health problem due to its high prevalence, increased mortality and major impact on quality of life and the economy^1^. End-stage renal disease (ESRD) is the fifth stage of CKD, which often requires renal replacement therapy, such as maintenance dialysis or kidney transplantation, to maintain life^2^. Hemodialysis patients are often accompanied by cognitive impairment (CI), the prevalence of which ranges from 30 to 76%, and even as high as 80.9% in China^3–5^. Although CI in CKD patients is usually manifested by the decline of executive function, the memory function of CKD patients, especially that of ESRD patients, also has a significant decline^5,6^.

At present, the mechanism of CI in patients with CKD is not clear. The "neurodegenerative hypothesis" associated with Alzheimer's disease (AD) is currently one of the main theories. Similar to AD, elevated serum Aβ levels have been found in patients with CKD^7^. In addition, an animal study has also certified that increased Aβ in the brain is associated with CKD^8^. Therefore, we speculate that AD-related pathophysiology may have occurred in the brain of patients with CKD prior to clinical diagnosis of CI.

The hippocampus is an important hub for memory and learning neural circuits. It has a significant contribution to memory and a variety of pathological changes, including ischemia, hypoxia, inflammation and toxin effects, etc., may cause neuronal damage to the hippocampus and lead to memory dysfunction^9–11^. Neuroimaging has been applied to characterize brain structural alterations in ESRD, advancing our understanding of ESRD-related neurological alterations. The decline of memory function in CKD patients may be related to structural or functional abnormalities of the hippocampus. Indeed, studies have reported smaller hippocampus volumes and impaired hippocampal network connectivity in patients with CKD, compared with healthy controls (HCs)^12,13^.

The hippocampus is a complex and heterogeneous structure consisting of several subfields with distinct histological features. Although the subfields are interconnected, they are relatively independent due to different functions. In addition, quantifying subfield volumes can significantly improve sensitivity to capture subtle atrophic patterns compared to overall hippocampal volume, thus providing more information in the early stages of disease^14^. Studies have shown that hippocampal subfields atrophy patterns vary in vulnerability to different neurological or psychiatric disorders, such as Alzheimer's disease (AD), Parkinson's disease, multiple sclerosis, and schizophrenia^15–18^. Due to the similarity between the occurrence of CKD-related cognitive impairment and the underlying pathological mechanism of AD, there is reason to suspect that patients with ESRD may also develop structural changes in the hippocampal subfields.

Cerebral laterality is a fundamental property of cortical functional organization^19^. The laterality of the human brain may serve as a neuroanatomical marker to predict cognitive function^20,21^. Likewise, the asymmetry of the hippocampus plays a special and important role in brain development. Studies have reported the increased asymmetry index (AI) of hippocampal subfields in various neuropsychiatric diseases, such as AD, diabetes, and depression^22–24^.

Nevertheless, to our best knowledge, there is no existing investigation considering hippocampal subfields atrophy or hippocampal asymmetry in ESRD, while only focusing on the whole hippocampus instead^12,25,26^. Furthermore, whether the structural alterations in the hippocampal subfields are associated with cognitive decline in ESRD patients remains unclear. Actually, exploring the hippocampal subfields, rather than treating the hippocampus as a separate entity, appears to be more conducive to understanding the mechanisms of memory decline in ESRD.

Therefore, this study aims to investigate the volumetric changes and asymmetry in the hippocampal subfields and their relationships with cognitive function and clinical characteristics. We hypothesized that specific hippocampal subfields volumes and hippocampal asymmetry were altered in ESRD patients compared to HCs. We also explored the relationships of these alterations with the memory decline as assessed by Rey's auditory verbal learning test (RAVLT)^27^ and with clinical characteristics.

## Materials and methods

### Participants

This prospective study was approved by the local ethics committee of the First Affiliated Hospital of Dalian Medical University and was performed in accordance with the relevant guidelines and regulations. Before the study, we obtained written informed consent from patients or their legal guardians. ESRD diagnosis was confirmed by nephrologist based on the kidney disease outcomes quality initiative (K/DOQI) classification. All patients were over 18 years old and followed maintenance hemodialysis (3–4 times per week) for at least three months. The exclusion criteria were: (a) history of psychiatric or neurological illness (schizophrenia, depression, AD, Parkinson's disease, etc.), severe brain trauma, brain tumor or any structural abnormalities showed on MRI examination; (b) kidney transplant recipients or acute renal failure (ARF); (c) any contraindications for magnetic resonance imaging (MRI) examination; (d) insufficient data on cognitive assessment; (e) image with motion artifacts.

Forty patients diagnosed with ESRD were prospectively enrolled between April 2021 and November 2021. According to the exclusion criteria, patients with poor MRI-image quality due to lack of contrast etc. (n = 2), with incomplete cognitive assessment (n = 1) and with claustrophobia (n = 4) were excluded. Therefore, 33 (16 men; 17 women; 60.73 ± 6.77 years) ESRD patients were enrolled in the final analysis. The underlying cause of ESRD in our study included glomerulonephritis (n = 10), hypertensive nephropathy (n = 10), diabetic nephropathy (n = 6), polycystic kidney disease (n = 5) and others (n = 2).

Over the same period, forty-six HCs (24 men; 22 women; 58.30 ± 8.06 years) with no previous history of neurological dysfunction were consecutively recruited from hospitals and nearby communities via digital advertising as a control group. The control group could represent the same source population as ESRD patients. All participants were right-handed.

### Demographic and clinical characteristics

Demographic data, including age, gender, education, body mass index (BMI), hypertension history, diabetes history and handedness were collected from all participants. Besides, we acquired blood biochemical tests from all participants within one week before MRI scanning, including hemoglobin (Hb), hematocrit (Hct), red blood cell (RBC), creatinine (Cre), uric acid (UA), serum urea (Urea), triglyceride (TG), homocysteine (HCY), serum calcium (Ca), serum phosphorus (P), albumin (Alb). In addition, all ESRD patients were tested for hypersensitive c-reactive protein (Hs-crp) and parathyroid hormone (PTH). Corrected calcium (cCa) and the single-pool kinetic transfer/volume urea measurements (spKt/V) were calculated. We also recorded dialysis duration, pre-and post-dialysis systolic blood pressure (SBP), diastolic blood pressure (DBP) and heart rate (HR) in ESRD patients.

### Neurocognitive assessments

All participants completed Beijing revised version Montreal Cognitive Assessment (MoCA) and RAVLT before MR data acquisition. MoCA can comprehensively and rapidly assess the overall cognitive function. RAVLT, which consists of learning and recall trials and a recognition memory trial, is a neurocognitive assessment tool to assess verbal memory^27^. After reading aloud a list of 15 nouns (List A) for five consecutive times, participants performed spontaneous recall (A1–A5). The first recall (A1) is related to short-term memory. The sum of the correct number of five recall tests is related to learning process (ΣA1–A5). Then, after the interference of another 15 nouns from List B, participants are required to recall the words from the initial list (A6, immediate recall). After 20 min (timed from the completion of List B recall), participants recall the words from List A again (A7, delayed recall). The recognition memory trial refers to providing a list of 50 words (including List A and List B), and participants are asked to check the words recognized from List A^28^.

### Magnetic resonance imaging acquisition

All MRI data was acquired on a 3.0 T MRI scanner (Ingenia CX, Philips Healthcare, Best, the Netherlands) equipped with a 32-channel phased-array head coil. High-resolution, T1-weighted (T1W) images were obtained using a three-dimensional multi-shot turbo field echo (MS-TFE) sequence with the following scan parameters: repetition time (TR)/echo time (TE) = 6.6/3.0 ms, flip angle (FA) = 12°, matrix size = 256 × 256, field of view (FOV) = 256 × 256 mm^2^, slices = 188, voxel size = 1 × 1 × 1 mm^3^.

### Hippocampal segmentation

The stable version 7.2.0 release (July 19, 2021) of the FreeSurfer software (https://surfer.nmr.mgh.harvard.edu) was applied for 3D-T1W image processing, involving the main automated pipeline of skull stripping, automated Talairach transformation, cortical and subcortical structure segmentation. We followed quality control and confirmed the accuracy of the segmentation results for each participant. The estimated total intracranial volume (eTIV) of each participant was also calculated and was used as a covariate in the statistical analysis.

**Figure 1:**
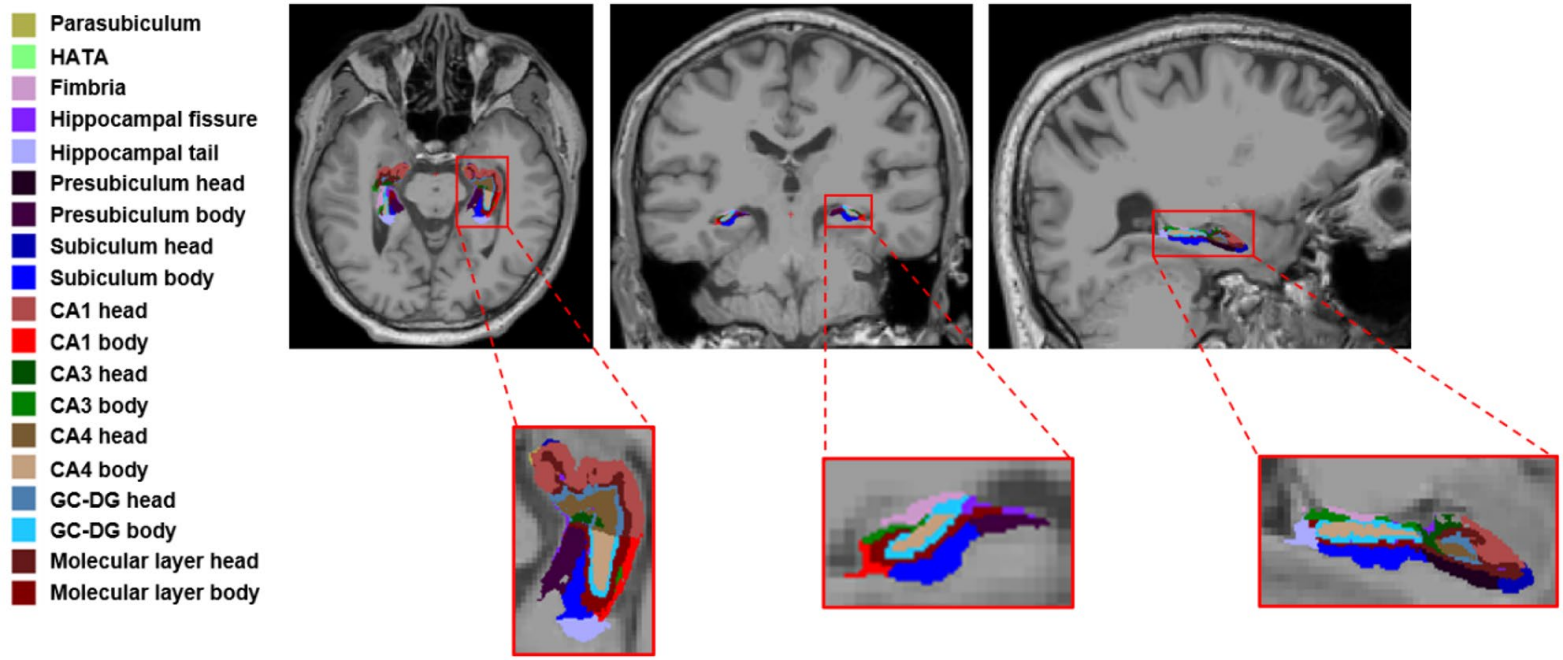
Hippocampal subfield segmentation. *CA* cornus ammonis, *GC-DG* granule cell layer of dentate gyrus, *HATA* hippocampus-amygdala transitional area.

The hippocampal module within the FreeSurfer based on T1W was applied to perform hippocampal subfields segmentation. The hippocampus was divided into 19 subfields: hippocampal tail, subiculum head, subiculum body, parasubiculum, presubiculum head, presubiculum body, CA1 head, CA3 head, CA4 head, GC-DG head, molecular layer head, HATA, CA1 body, CA3 body, CA4 body, GC-DG body, molecular layer body, fimbria and hippocampal fissure (Fig. 1).

### Asymmetry index (AI)

As previous studies described^23^, we used the following formula to quantify the asymmetry of hippocampal subfields:

$$AI= \frac{|Left-Right|}{|Left+Right|}\times 100\%.$$

### Statistical analysis

Statistical analysis was conducted using SPSS version 22.0. The K-S test was used to test the normality of the data. Normally and non-normally distributed demographic characteristics were analyzed by two-sample t tests and Mann–Whitney U tests, respectively. Chi-squared (χ^2^) tests was performed for proportions.

The volumes and AI of the hippocampal subfields between ESRD and HCs were compared using covariance analysis (ANCOVA) with age, gender, education, eTIV as covariates. In addition, it was reported that hypertension and diabetes also had effect on the volume of hippocampal subfields^29,30^. Therefore, in our study, we also included the history of hypertension and diabetes as covariates for statistical analysis. The Benjamini–Hochberg false discovery rate (FDR) correction was applied for multiple testing. In addition, ROC curve analysis was performed to evaluate the sensitivity and specificity of subfields volume in discriminating ESRD from HCs. Only volumes of hippocampal subfields showing differences will be used for ROC analysis.

To investigate the relationship between atrophied hippocampal subfield volumes and memory performance as well as blood biochemical tests, partial correlation analysis was adopted, with age, gender, education, eTIV, hypertension and diabetes history as covariables. Statistical tests were two tailed, and the significant threshold was set at *p* < 0.05.

## Results

### Demographic and clinical characteristics

The demographic and clinical characteristics of the ESRD patients and HCs are summarized in the Table 1. Gender, age, BMI and years of education between the two groups showed no statistical difference (*p* > 0.05). The incidence of hypertension in ESRD patients is higher (Z = 24.416, *p* < 0.001). ESRD patients had lower Hb (t = − 11.451, *p* < 0.001), Hct (t = − 12.266, *p* < 0.001), RBC (t = − 13.025, *p* < 0.001) and Alb (t = − 12.242, *p* < 0.001) and higher Cre (t = 38.89, *p* < 0.001), UA (t = 6.547, *p* < 0.001), Urea (Z = − 6.428, *p* < 0.001), HCY (Z = − 7.157, *p* < 0.001), P (t = 9.721, *p* < 0.001) and cCa (t = 3.523, *p* = 0.001) than HCs.

**Table 1:** Demographic and clinical characteristics.

	ESRD (n = 33)	HCs (n = 46)	t/χ^2^/Z	*p* value
Gender (M/F)	16/17	24/22	0.821	0.462
Age, years	60.73 ± 6.77	58.30 ± 8.06	1.407	0.164
BMI, kg/m^2^	23.60 ± 2.42	24.40 ± 2.42	− 1.455	0.150
Education, years, M (IQR)	9 (9, 12)	12 (9, 15)	− 1.926	0.054
Hypertension, n (%)	24 (72.73)	8 (17.39)	24.416	< 0.001*
Diabetes Mellitus, n (%)	10 (30.30)	7 (15.22)	2.589	0.164
Dialysis duration, year	7 (2, 15)	–		
Hb, g/L	110.58 ± 8.12	143.27 ± 14.96	− 11.451	< 0.001*
Hct, L/L	33.96 ± 2.47	43.22 ± 3.80	− 12.266	< 0.001*
RBC, 10^12^/L	3.65 ± 0.33	4.79 ± 0.42	− 13.025	< 0.001*
Cre, umol/L	934.91 ± 156.44	63.31 ± 13.95	38.89	< 0.001*
UA, umol/L	435.21 ± 76.02	315.96 ± 83.98	6.547	< 0.001*
Urea, mmol/L	26.35 (22.12, 31.24)	5.12 (4.39, 6.33)	− 6.428	< 0.001*
TG, mmol/L	1.41 (1.1, 2.16)	1.18 (0.86, 1.50)	− 1.513	0.130
HCY, umol/L	22.19 (18.15, 28.71)	9.25 (7.725, 11.915)	− 7.157	< 0.001*
Ca, mmol/L	2.27 ± 0.15	2.30 ± 0.09	− 0.898	0.374
P, mmol/L	1.92 ± 0.45	1.08 ± 0.19	9.721	< 0.001*
Alb, g/L	38.14 ± 2.38	44.83 ± 2.49	− 12.242	< 0.001*
cCa, mmol/L	2.31 ± 0.16	2.20 ± 0.08	3.523	0.001*
spKt/V	1.47 (1.22, 1.63)	–		
Hs-crp, mg/L	2.2 (0.97, 5.27)	–		
PTH, pg/ml	287.10 (136.35, 526.15)	–		
Pre-dialysis SBP, mmHg	152.20 ± 20.02	–		
Post-dialysis SBP, mmHg	144.50 ± 20.32	–		
Pre-dialysis DBP, mmHg	79.83 ± 9.95	–		
Post-dialysis DBP, mmHg	80.67 ± 7.65	–		
Pre-dialysis HR, bpm	72.87 ± 8.42	–		
Post-dialysis HR, bpm	71.93 ± 9.03	–		

*HC* health controls, *ESRD* end stage renal disease, *BMI* body mass index, *Hb* hemoglobin, *Hct* hematocrit, *RBC* red blood cell, *Cre* creatinine, *UA* uric acid, *Urea* serum urea, *TG* triglyceride, *HCY* homocysteine, *Ca* serum calcium, *P* serum phosphorus, *Alb* albumin, *cCa* corrected calcium, *spKt/V* single-pool kinetic transfer/volume urea measurements, *Hs-crp* hypersensitive c-reactive protein, *PTH* parathyroid hormone, *SBP* systolic blood pressure, *DBP* diastolic blood pressure, *HR* heart rate, *M* median, *n* number, *IQR* interquartile range.

Data are given as mean ± standard deviation (SD), n (%), or M (IQR).

*Represents the statistical difference between the two groups, p < 0.05.

In terms of neurocognitive assessments (Table 2), compared to HCs, ESRD group had poorer performances on MoCA (Z = − 2.130, *p* = 0.033, df = 77), RAVLT short-term memory (Z = − 2.496, *p* = 0.013, df = 77) and RAVLT delayed recall (Z = − 2.016, *p* = 0.044, df = 77), while no significant differences were observed in other RAVLT scores (*p* > 0.05).

**Table 2:** Neuropsychological assessment between ESRD and HCs.

	ESRD (n = 33)	HCs (n = 46)	t/Z	*p* value
MoCA, M (IQR)	26 (22.5, 27)	27 (23, 28)	− 2.130	0.033*
Visuospatial/executive, M (IQR)	4 (3, 4)	5 (4, 5)	− 4.703	< 0.001*
Naming, M (IQR)	3 (3, 3)	3 (3, 3)	− 1.802	0.072
Attention, M (IQR)	5 (4, 5)	6 (5, 6)	− 4.043	< 0.001*
Language, M (IQR)	2 (1, 3)	2.5 (2, 3)	− 2.613	0.009*
Abstraction, M (IQR)	1(0, 2)	2 (1, 2)	− 2.654	0.008*
Delayed Recall, M (IQR)	4 (3, 5)	3 (1, 4)	− 2.331	0.020*
Orientation, M (IQR)	6 (6, 6)	6 (6, 6)	− 0.961	0.337
RAVLT, short-term memory	4 (3.5, 5)	5 (4, 6)	− 2.496	0.013*
RAVLT, learning process	40.39 ± 9.31	43.93 ± 8.52	− 1.752	0.084
RAVLT, immediate recall	8 (6.5, 11.5)	9.5 (7, 11.25)	− 1.256	0.209
RAVLT, delayed recall	7 (6, 10)	9 (7, 11)	− 2.016	0.044*
RAVLT, recognition	14 (13, 14.5)	14 (13, 15)	− 0.584	0.559

*HC* health controls, *ESRD* end stage renal disease, *BMI* body mass index, *M* median, *n* number, *IQR* interquartile range, *RAVLT* Rey auditory verbal learning test.

Data are given as mean ± standard deviation (SD), n (%), or M (IQR).

*Represents the statistical difference between the two groups, *p* < 0.05.

### Hippocampal subfields alterations in ESRD

**Figure 2:**
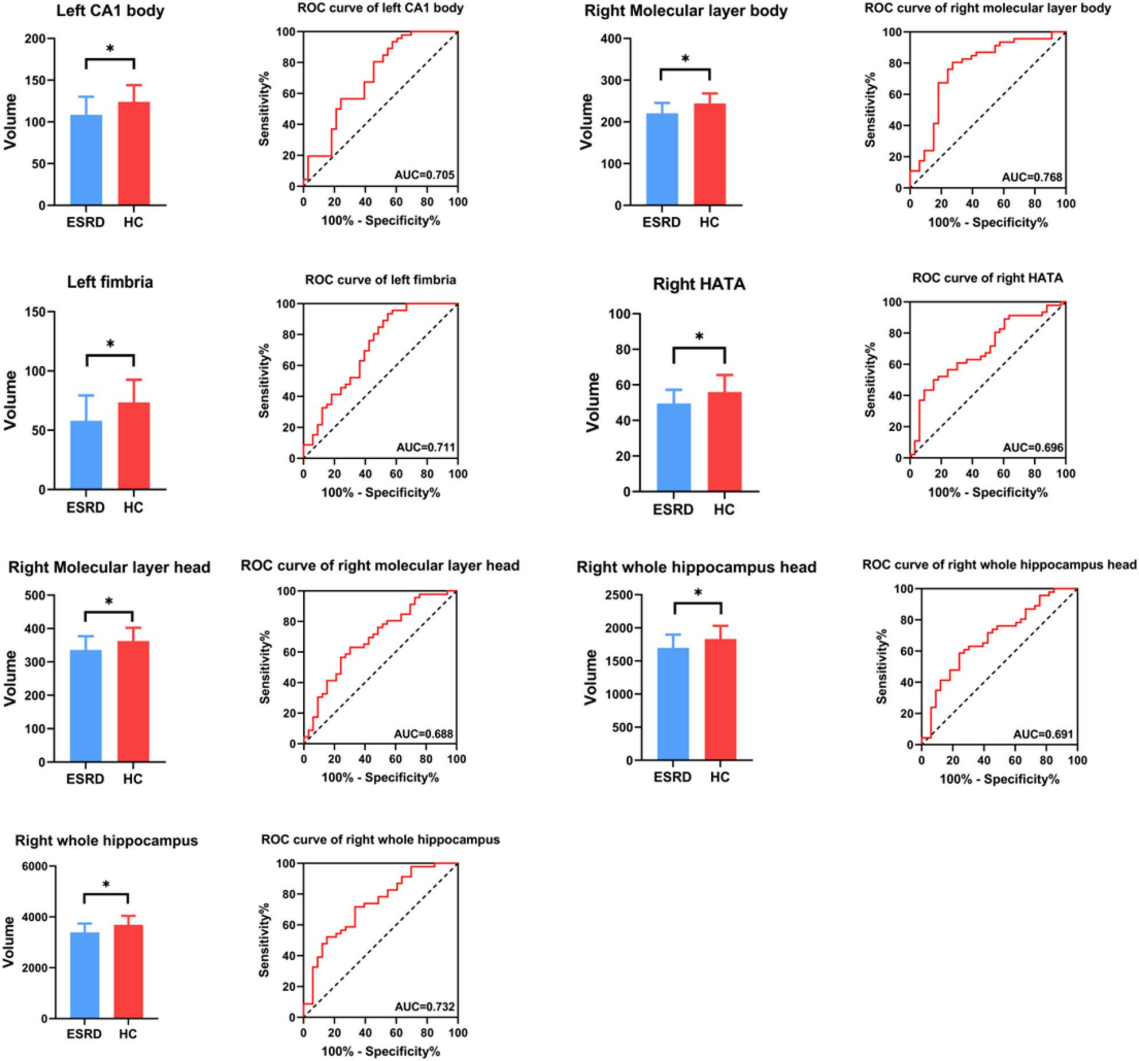
Volumes of the whole hippocampus and subfields showed significant group difference in ESRD and HCs. *represents the statistical difference between the two groups, correction adjusted *p* value (*q* value) < 0.05. ROC curves analysis based on the left CA1 body, left fimbria, right molecular layer head, right molecular layer body, right HATA, right whole hippocampal head and right whole hippocampal volumes. *CA* cornus ammonis, *HATA* hippocampus-amygdala transitional area.

The volumes of the whole and subfields of hippocampal are summarized in Table 3 and Fig. 2. Concerning the whole structures, compared to HCs, the volume of the right hippocampus in ESRD was significantly reduced (F = 10.732, *p* = 0.002, *q* = 0.024, FDR corrected). For the subfields, ESRD patients had reduced volumes in the left CA1 body (F = 7.875, *p* = 0.007, *q* = 0.041, df = 1), left fimbria (F = 8.600, *p* = 0.005, *q* = 0.040, df = 1), right molecular layer head (F = 8.225, *p* = 0.005, *q* = 0.040, df = 1), right molecular layer body (F = 10.958, *p* = 0.001, *q* = 0.024, df = 1), right HATA (F = 10.923, *p* = 0.002, *q* = 0.024, df = 1).

**Table 3:** Comparison of hippocampus and hippocampal subfields volumes (mm^3^) between ESRD and HCs.

	ESRD (n = 33)	HC (n = 46)	F	*p* value	FDR *q* value
Left					
Hippocampal tail	519.87 ± 71.12	568.33 ± 71.41	5.322	0.024	0.071
Subiculum body	254.55 ± 23.13	259.65 ± 28.54	0.356	0.553	0.593
CA1 body	108.36 ± 21.74	123.95 ± 20.07	7.875	0.007	0.041*
Subiculum head	193.25 ± 29.71	200.15 ± 26.35	0.704	0.404	0.468
Hippocampal fissure	155.93 ± 41.81	140.92 ± 26.73	2.155	0.147	0.215
Presubiculum head	136.31 ± 19.40	145.45 ± 15.48	4.218	0.044	0.095
CA1 head	491.26 ± 65.37	524.14 ± 66.01	3.343	0.072	0.117
Presubiculum body	177.71 ± 21.90	183.69 ± 28.83	0.058	0.810	0.829
Parasubiculum	60.88 ± 19.68	61.97 ± 12.90	0.182	0.671	0.703
Molecular layer head	322.99 ± 40.62	341.80 ± 37.76	3.658	0.060	0.106
Molecular layer body	216.06 ± 26.46	233.57 ± 23.25	4.795	0.032	0.083
GC-DG head	144.30 ± 21.44	154.98 ± 23.00	3.390	0.070	0.117
CA3 body	89.46 ± 17.54	90.84 ± 14.36	0.020	0.889	0.889
GC-DG body	136.17 ± 17.24	138.20 ± 12.78	0.492	0.485	0.534
CA4 head	123.75 ± 17.40	130.22 ± 17.85	2.267	0.137	0.207
CA4 body	123.52 ± 15.70	123.67 ± 10.53	1.098	0.298	0.366
Fimbria	57.92 ± 21.44	73.45 ± 19.13	8.600	0.005	0.040*
CA3 head	116.31 ± 21.26	120.72 ± 22.80	0.920	0.341	0.405
HATA	48.53 ± 9.65	54.07 ± 9.48	2.025	0.159	0.226
Whole hippocampus body	1163.76 ± 121.41	1223.34 ± 113.53	1.374	0.245	0.327
Whole hippocampus head	1637.57 ± 203.84	1733.50 ± 184.17	4.002	0.049	0.095
Whole hippocampus	3321.20 ± 355.46	3525.17 ± 308.61	4.897	0.030	0.083
Right					
Hippocampal tail	533.81 ± 63.25	591.88 ± 84.85	6.169	0.015	0.064
Subiculum body	245.45 ± 25.23	258.29 ± 27.62	1.267	0.264	0.342
CA1 body	122.55 ± 17.45	136.49 ± 20.09	5.629	0.020	0.064
Subiculum head	199.51 ± 29.09	211.53 ± 23.69	2.998	0.088	0.138
Hippocampal fissure	165.60 ± 31.92	150.22 ± 34.98	1.092	0.300	0.366
Presubiculum head	133.06 ± 16.85	143.06 ± 13.73	5.869	0.018	0.064
CA1 head	522.73 ± 68.26	563.74 ± 68.65	4.678	0.034	0.083
Presubiculum body	149.32 ± 19.55	162.40 ± 31.23	0.640	0.426	0.481
Parasubiculum	52.31 ± 12.58	57.86 ± 14.33	3.665	0.060	0.106
Molecular layer head	336.18 ± 40.79	362.80 ± 39.68	8.225	0.005	0.040*
Molecular layer body	220.63 ± 24.76	244.23 ± 24.08	10.958	0.001	0.024*
GC-DG head	153.48 ± 24.11	165.54 ± 22.84	6.099	0.016	0.064
CA3 body	100.04 ± 14.82	102.63 ± 15.75	1.673	0.200	0.275
GC-DG body	136.38 ± 16.90	147.47 ± 15.21	5.712	0.020	0.064
CA4 head	131.11 ± 19.17	138.98 ± 16.36	6.007	0.017	0.064
CA4 body	125.30 ± 14.99	132.11 ± 13.34	4.018	0.049	0.095
Fimbria	62.44 ± 23.88	84.53 ± 31.07	4.022	0.049	0.095
CA3 head	121.16 ± 21.44	129.91 ± 20.33	4.158	0.045	0.095
HATA	49.30 ± 7.81	55.99 ± 9.51	10.923	0.002	0.024*
Whole hippocampus body	1162.10 ± 121.99	1264.16 ± 129.17	6.212	0.015	0.064
Whole hippocampus head	1698.85 ± 199.18	1832.47 ± 198.18	8.993	0.004	0.040*
Whole hippocampus	3394.76 ± 343.98	3565.80 ± 376.06	10.732	0.002	0.024*

Adjusted age, gender, education, eTIV, hypertension and diabetes history.

Data are given as mean ± standard deviation (SD).

*HC* health controls, *ESRD* end stage renal disease, *FDR* false discovery rate, *CA* cornus ammonis, *GC-DG* granule cell layer of dentate gyrus, *HATA* hippocampus-amygdala transitional area.

*Represents the statistical difference between the two groups, *p* value (*q* value) < 0.05.

For the asymmetry of hippocampal subfields, there was no significant difference between the two groups (Supplementary Material, Table S1).

### ROC curve analysis

Table 4 and Fig. 2 presents the ROC curve analysis results of the whole hippocampus and subfields volumes differentiating between ESRD and HCs. The results demonstrated that the classifications based on the right molecular layer body (AUC = 0.768) resulted in higher performance than the right whole hippocampal volume (AUC = 0.732) for AUC. Besides, the left fimbria also had well performance on differentiating ESRD patients from HCs (AUC = 0.711). Further diagnostic analysis showed that the right molecular layer body discriminated ESRD from HCs with a sensitivity of 80.43% and specificity of 72.73% at the largest Youden index, with the left fimbria a sensitivity of 93.48% and specificity of 45.45%.

**Table 4:** ROC analysis for differentiating ESRD patients from HCs.

Hippocampus and subfields	AUC	Sensitivity (%)	Specificity (%)	Cutoff point (mm^3^)
Left CA1 body	0.705	93.48	42.42	98.26
Left fimbria	0.711	93.48	45.45	50.02
Right molecular layer head	0.688	63.04	69.7	352.00
Right molecular layer body	0.768	80.43	72.73	228.30
Right HATA	0.696	50.00	84.85	54.99
Right whole hippocampus head	0.691	58.7	75.76	1796
Right whole hippocampus	0.732	71.74	66.67	3477

*ROC* receiver operating characteristic, *AUC* area under curve, *HC* health controls, *ESRD* end stage renal disease, *CA* cornus ammonis, *HATA* hippocampus-amygdala transitional area.

### Correlation analysis of hippocampal subfields volumes and RAVLT

**Figure 3:**
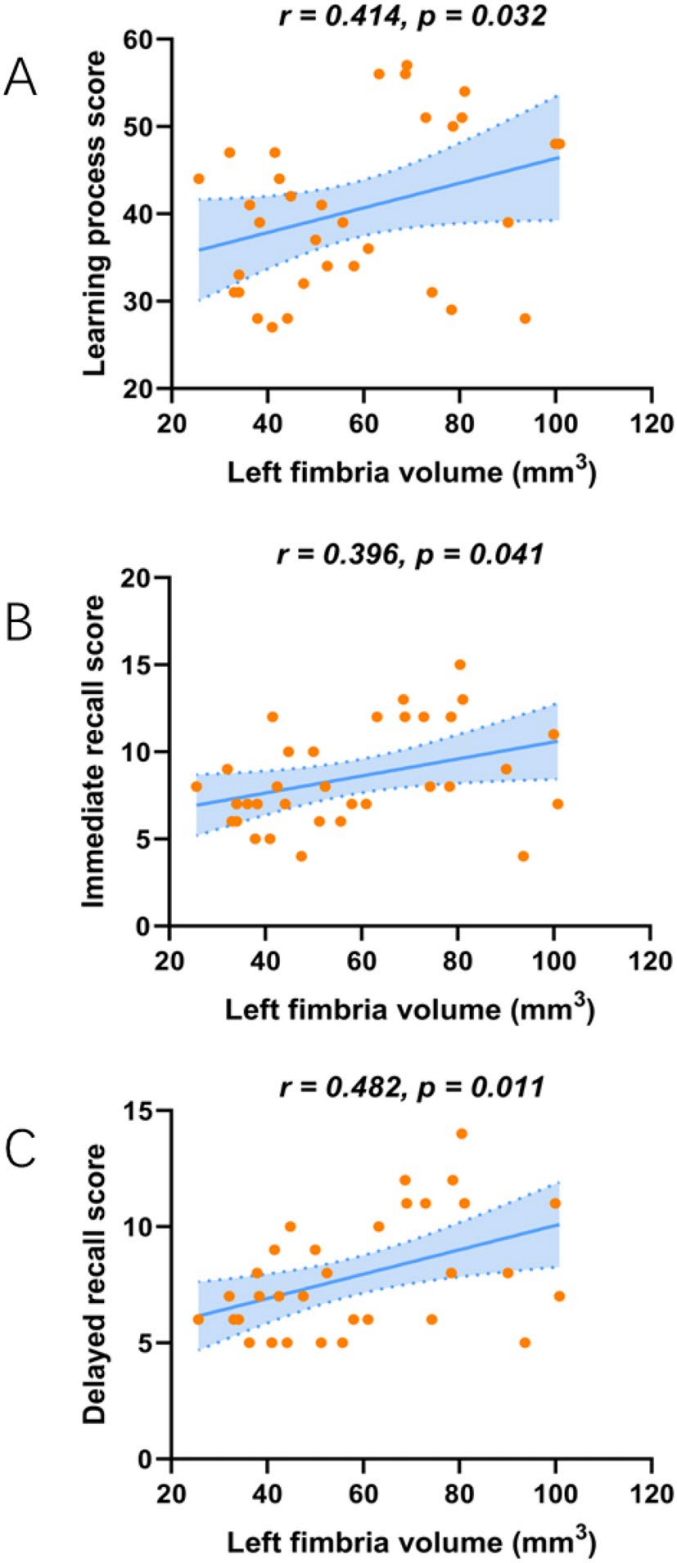
Volume of the left fimbria was correlated with learning process scores (*r* = 0.414, *p* = 0.032) (**A**), immediate recall scores (*r* = 0.396, *p* = 0.041) (**B**) and delayed recall scores (*r* = 0.482, *p* = 0.011) (**C**). Adjusted age, gender, education, eTIV and hypertension and diabetes history.

Figure 3 shows the results of partial correlation analysis between the volumes of atrophic hippocampal subfields and RAVLT in the ESRD group, controlling for age, gender, education, eTIV, hypertension and diabetes history as covariables. The volume of the left fimbria was positively correlated with learning process score (*r* = 0.414, *p* = 0.032), immediate recall score (*r* = 0.396, *p* = 0.041) and delayed recall score (*r* = 0.482, *p* = 0.011). No significant correlations were found between other hippocampal subfield volumes and RAVLT (*p* > 0.05).

### Correlation analysis of hippocampal subfields volumes and blood biochemical tests

**Figure 4:**
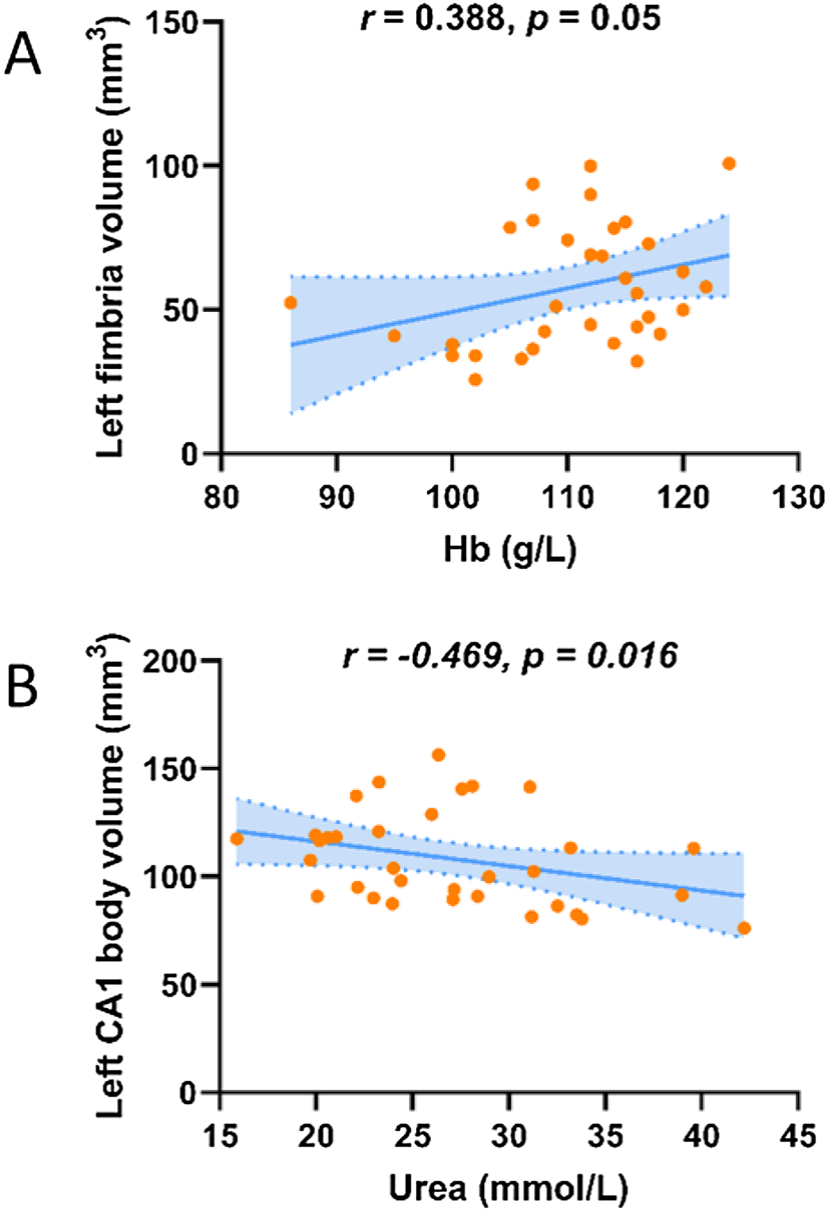
(**A**) Hb (*r* = 0.388, *p* = 0.05) was positively correlated with the volume of the left fimbria. (**B**) Urea was negatively correlated with the left CA body volume (*r* = − 0.469, *p* = 0.016). Adjusted age, gender, education, eTIV and hypertension and diabetes history. *Hb* hemoglobin, *Urea* serum urea.

In the ESRD group, partial correlation analysis showed that the volume of the left fimbria had a positive correlation trend with Hb (*r* = 0.388, *p* = 0.050), the left CA1 body volume had a negative correlation with Urea (*r* = − 0.469, *p* = 0.016) (Fig. 4). No significant correlations were found between other blood biochemical tests and the reduced hippocampal subfield volumes (*p* > 0.05).

## Discussion

In this study, we first investigated the volumes and asymmetry alterations of hippocampal subfields in patients with ESRD as well as their associations with memory ability and blood biochemical tests. We found the following points: (1) ESRD patients had impaired memory function, mainly in short-term memory and delayed recall. (2) In ESRD patients, the five atrophied hippocampal subfields were the left CA1 body, left fimbria, right molecular layer head, right molecular layer body and right HATA. (3) The atrophy of left fimbria was related to learning process, immediate recall and delayed recall. (4) Anemia and Urea may be associated with the atrophy of the left fimbria and left CA1 body.

Previous studies have demonstrated the associations of hippocampal structure and function abnormalities with ESRD^12,13^. In our research, we observed reduced volume of the right whole hippocampus, consistent with the previous findings. In addition, unlike previous studies that focused on the hippocampus as a whole, we first analyzed the volume changes of the hippocampal subfields in ESRD patients and found the differences with HCs after controlling for age, sex, education, eTIV, hypertension and diabetes history. Furthermore, the right molecular layer body volume significantly outperformed the whole right hippocampal volume in discriminating between ESRD and HCs could also illustrate the importance of focusing on the changes in hippocampal subfields.

The hippocampus plays a crucial role in both information processing and episodic and spatial memory. Anatomically, the atrophic structures of the ESRD patients in our study, including the molecular layers, CA1, and HATA, are located on the lateral portions of the hippocampus. The hippocampal CA1 is a major component of the memory circuit in the medial temporal lobe and is a key region for memory encoding and formation^31^. Anatomical and physiological studies have shown that the information conversion circuit of the hippocampus starts from the dentate gyrus and finally regulates the activity of the hippocampal circuit and learning and memory through the projection pathway between CA1 and the subiculum^32,33^. Our study showed that compared with HCs, the left CA1 body of ESRD patients was smaller and the result was also supported by previous animal experiment, which reported that neuronal vulnerability of hippocampal CA1 was associated with memory dysfunction after chronic renal failure^34^. The molecular layer is situated between the subiculum and the fissure, which consists of part of the subiculum and CA fields. The reduced number of synapses in the molecular layer of hippocampus may affect information transmission between pyramidal cells and interneurons, which in turn affects the connectivity between subfields, ultimately leading to memory impairment^23^. Studies have certified that the number of synapses in the molecular layer correlated with cognitive performance in AD or mild cognitive impairment (MCI)^35^. However, this study has not yet found any correlation between CA1 or molecular layer atrophy and memory function, and we consider that it may be due to the small sample size, or that some of our ESRD patients have acceptable memory function, which masks this correlation. Subsequent studies should be conducted by expanding the sample size and grouping by cognitive impairment.

HATA, located in the medial portion of the hippocampus, is closely connected to the amygdala and is part of the hippocampus-amygdala pathway^36^. The fimbria is a white matter structure that forms part of the fornix and projects information to the amygdala^36^. Previous studies showed that the HATA and fimbria are involved in visuospatial function and object discrimination by modulating the amygdala-hippocampal pathway^37^. Therefore, we speculate that atrophy of these regions may cause damage to the fornix or the hippocampus-amygdala pathway, and may be a biomarker of visuospatial dysfunction in ESRD patients. In addition, Yu et al. found that decreased volume of hippocampus predicted deterioration of fornix microstructure^38^. Our previous study identified the vulnerability of the fornix microstructure in ESRD patients^39^. Therefore, based on the location of the fimbria in the hippocampus and the anatomical relationship with the fornix, we speculate that the change of fimbria volume may be related to the microstructure of the fornix. However, this speculation needs to be verified. At present, studies on the atrophy of fimbria are still inconsistent. Mixed studies suggest fimbria atrophy or retention in MCI and AD^40–42^, and large-scale clinical studies are required for unified conclusion. We found that learning process, immediate recall and delayed recall scores were closely related to the left fimbria volume, which consistent with previous research^43^. Animal experiments also showed the relationship between fimbria-fornix damage and immediate recall impairment^44^.

Based on the result that decreased hemoglobin correlated with smaller left fur volume in our study, we hypothesized that anemia may promote left fimbria atrophy. It has been reported that most hemodialysis patients suffer from renal anemia. In ESRD patients, renal anemia is incurable due to persistent impairment of renal function and decreased production of erythropoietin^45^. Renal anemia can lead to hypoxia, and the hippocampus is particularly vulnerable to hypoxia^46^, which may cause local atrophy. However, the impact of anemia on the hippocampal subfields is still a complex issue, and larger sample study is still required. Besides, Urea was found to be correlated with the atrophy of left CA1 body. Kidney failure can cause abnormal accumulation of a variety of toxic substances, such as urea, creatinine and uric acid^47^. These neurotoxic substances may also be present in the cerebrospinal fluid of patients with kidney failure, inducing glial cells and neurons to atrophy and die and leading to brain atrophy^48^. Wang et al. also found the association between elevated serum urea levels and brain atrophy^49^. Therefore, we speculate that serum urea may also lead to atrophy of the hippocampal subfields. However, the pathophysiological mechanism needs to be further explored.

Previously, brain asymmetry is a key morphological indicator reflecting developmental and pathological alterations^20^. The hippocampus is one of the asymmetric regions in the brain, especially in neurodegenerative diseases, such as AD^50^. In this study, we found that the right hippocampus had more volume loss subfields compared to the left hippocampus. Previous study on posterior cortical atrophy also found hippocampal atrophy was predominant in the right hemisphere^51^. But our study did not find any difference of AI between the two groups. We consider this to be the reason for the similar atrophy of the left and right hippocampus after cognitive impairment in ESRD patients.

Some limitations should be considered in this study. First, the small sample size in the prospective study is the main limitation, and further large sample size researches are still needed; Second, based on the cross-sectional study design, we were unable to assess the changes of the hippocampal subfield volumes in ESRD patients, and longitudinal follow-up studies are required; Third, we did not consider the etiology of ESRD patients and confounding effects of etiology may be existed and affect cognitive performance. Forth, although the FreeSurfer automated segmentation procedure within the spatial resolution of 1mm3 isotropic in 3D-T1 image has been found to be a test–retest reliable method^52^, the segmentation results of the hippocampal subfields should also be validated at higher resolution in the future.

## Conclusions

In conclusion, we found reduced hippocampal subfield and whole hippocampal volumes in ESRD patients. In particularly, the reduction in the left fimbria was closely related to memory function. Besides, anemia and Urea may be associated with the atrophy of the left fimbria and CA1 body. We believe the findings of this work will contribute to understanding the mechanisms of memory decline in ESRD.

## Supplementary Information

Supplementary Table S1.

## Data Availability

All data included in this study are available upon request by contact with the corresponding author.

## Abbreviations

AI: Asymmetric index
CKD: Chronic kidney disease
ESRD: End stage renal disease
eTIV: Estimated total intracranial volume
FDR: False discovery rate
HC: Healthy control
IQR: Interquartile range
MRI: Magnetic resonance imaging
MoCA: Montreal Cognitive Assessment
RAVLT: Rey's auditory verbal learning test
ROC: Receiver operating characteristic
