# Supplementary material for: Relations of hippocampal subfields atrophy patterns with memory and biochemical changes in end stage renal disease
Source: Sci Rep. 2023 Feb 20;13:2982. doi: 10.1038/s41598-023-29083-0 (PMC9941083; doi:10.1038/s41598-023-29083-0)
Supplement: Supplementary file 1 — Supplementary Table S1. [file 41598_2023_29083_MOESM1_ESM.docx]

**Table S1. Comparison of the asymmetry of hippocampal subfields (%)**

**between ESRD and HCs**

|  | **ESRD**  **(n=33)** | **HC**  **(n=46)** | **F** | ***p* value** | **FDR *q* value** |
| --- | --- | --- | --- | --- | --- |
| Hippocampal tail | 3.81±2.66 | 3.94±2.81 | 1.772 | 0.19 | 0.82 |
| Subiculum body | 3.42±2.36 | 3.01±2.65 | 0.116 | 0.73 | 0.82 |
| CA1 body | 7.97±6.54 | 7.86±5.93 | 0.322 | 0.57 | 0.82 |
| Subiculum head | 4.64±3.59 | 5.20±3.29 | 0.174 | 0.68 | 0.82 |
| Hippocampal fissure | 6.70±5.54 | 6.23±5.19 | 0.199 | 0.66 | 0.82 |
| Presubiculum head | 4.54±3.71 | 3.87±3.31 | 0.153 | 0.70 | 0.82 |
| CA1 head | 3.54±2.75 | 3.85±2.44 | 0.087 | 0.77 | 0.82 |
| Presubiculum body | 8.86±5.92 | 6.85±3.50 | 0.321 | 0.57 | 0.82 |
| Parasubiculum | 11.63±9.22 | 7.37±5.23 | 2.715 | 0.10 | 0.82 |
| Molecular layer head | 2.89±2.39 | 3.17±2.04 | 0.228 | 0.63 | 0.82 |
| Molecular layer body | 2.95±2.17 | 3.32±2.28 | 0.014 | 0.91 | 0.91 |
| GC-DG head | 4.44±2.46 | 4.45±3.22 | 0.339 | 0.56 | 0.82 |
| CA3 body | 8.21±6.95 | 6.92±5.21 | 0.711 | 0.40 | 0.82 |
| GC-DG body | 3.44±2.58 | 4.36±3.18 | 0.833 | 0.36 | 0.82 |
| CA4 head | 4.47±2.67 | 4.03±3.49 | 1.512 | 0.22 | 0.82 |
| CA4 body | 3.66±2.62 | 4.48±3.07 | 0.073 | 0.79 | 0.82 |
| Fimbria | 15.68±10.59 | 11.97±11.74 | 2.737 | 0.10 | 0.82 |
| CA3 head | 4.75±3.99 | 5.08±4.34 | 0.857 | 0.36 | 0.82 |
| HATA | 6.79±5.64 | 6.26±4.74 | 0.747 | 0.39 | 0.82 |

Adjusted age, gender, education, eTIV, hypertension and diabetes history.

Data are given as mean ± standard deviation (SD).

HC, health controls; ESRD, end stage renal disease; FDR, false discovery rate; CA, cornus ammonis; GC-DG, granule cell layer of dentate gyrus; HATA, hippocampus-amygdala transitional area.
